# Supplementary material for: Obstructive Sleep Apnoea in Children with Down Syndrome: A Multidisciplinary Approach
Source: J Pers Med. 2022 Dec 28;13(1):71. doi: 10.3390/jpm13010071 (PMC9862921; doi:10.3390/jpm13010071)
Supplement: Supplementary file 1 [file jpm-13-00071-s001.zip › Supplementary file S1.pdf]

CSHQ-IT
(Età prescolare e scolare)

Le seguenti affermazioni riguardano le abitudini del sonno del suo bambino e le possibili difficoltà collegate al suo sonno.
Nel rispondere alle domande pensi a ciò che è successo al suo bambino durante la scorsa settimana. Se la scorsa settimana è stata insolita per un motivo particolare (ad esempio il bambino ha avuto un'otite e non ha dormito bene o il televisore era rotto), prenda in considerazione la settimana tipo più recente.
Risponda DI SOLITO se qualcosa si verifica 5 o più volte in una settimana; risponda QUALCHE VOLTA se si verifica 2-4 volte in una settimana; risponda RARAMENTE se non si verifica mai o una volta nel corso di una settimana. La preghiamo anche di indicare se le caratteristiche del sonno rappresentano un problema cerchiando "Si", "No", o "Non pertinente (N/P)".

Ora in cui il bambino va a letto
Scriva l'orario in cui di solito il bambino va a dormire la sera:_____

1. Il bambino va a letto sempre alla stessa ora di sera

Di solito (5-7)☐ Qualche volta (2-4)☐ Raramente (0-1) ☐ Problema? Si No N/P

2. Il bambino si addormenta entro 20 minuti dopo essere andato a letto

Di solito (5-7)☐ Qualche volta (2-4)☐ Raramente (0-1) ☐ Problema? Si No N/P

3. Il bambino si addormenta da solo nel proprio letto

Di solito (5-7)☐ Qualche volta (2-4)☐ Raramente (0-1) ☐ Problema? Si No N/P

4. Il bambino si addormenta nel letto dei genitori o dei fratelli

Di solito (5-7)☐ Qualche volta (2-4)☐ Raramente (0-1) ☐ Problema? Si No N/P

5. Il bambino ha bisogno dei genitori nella stanza per addormentarsi

Di solito (5-7)☐ Qualche volta (2-4)☐ Raramente (0-1) ☐ Problema? Si No N/P

6. Il bambino fa i capricci al momento di andare a dormire (piange, rifiuta di stare a letto, ecc.)

Di solito (5-7)☐ Qualche volta (2-4)☐ Raramente (0-1) ☐ Problema? Si No N/P

7. Il bambino ha paura di dormire al buio

Di solito (5-7)☐ Qualche volta (2-4)☐ Raramente (0-1) ☐ Problema? Si No N/P

8. Il bambino ha paura di dormire da solo

Di solito (5-7)☐ Qualche volta (2-4)☐ Raramente (0-1) ☐ Problema? Si No N/P

Comportamento durante il sonno
Tempo abituale di sonno del bambino ogni giorno: ______ore e ______minuti
(considerando sonno notturno e pisolini)

9. Il bambino dorme troppo poco

Di solito (5-7)☐ Qualche volta (2-4)☐ Raramente (0-1) ☐ Problema? Si No N/P

10. Il bambino dorme la giusta quantità di ore

Di solito (5-7)☐ Qualche volta (2-4)☐ Raramente (0-1) ☐ Problema? Si No N/P

11. Il bambino dorme per lo stesso numero di ore ogni giorno

Di solito (5-7)☐ Qualche volta (2-4)☐ Raramente (0-1) ☐ Problema? Si No N/P

12. Il bambino bagna il letto di notte

Di solito (5-7)☐ Qualche volta (2-4)☐ Raramente (0-1) ☐ Problema? Si No N/P

13. Il bambino parla durante il sonno

Di solito (5-7)☐ Qualche volta (2-4)☐ Raramente (0-1) ☐ Problema? Si No N/P

14. Il bambino è inquieto e si muove molto durante il sonno

Di solito (5-7)☐ Qualche volta (2-4)☐ Raramente (0-1) ☐ Problema? Si No N/P

15. Il bambino è sonnambulo durante la notte

Di solito (5-7)☐ Qualche volta (2-4)☐ Raramente (0-1) ☐ Problema? Si No N/P

16. Il bambino va nel letto di qualcun altro durante la notte (genitori, fratello, sorella, ecc.)

Di solito (5-7)☐ Qualche volta (2-4)☐ Raramente (0-1) ☐ Problema? Si No N/P

17. Il bambino digrigna i denti durante il sonno (il suo dentista potrebbe avergliene parlato)

Di solito (5-7)☐ Qualche volta (2-4)☐ Raramente (0-1) ☐ Problema? Si No N/P

18. Il bambino russa rumorosamente

Di solito (5-7)☐ Qualche volta (2-4)☐ Raramente (0-1) ☐ Problema? Si No N/P

19. Il bambino sembra smettere di respirare durante il sonno

Di solito (5-7)☐ Qualche volta (2-4)☐ Raramente (0-1) ☐ Problema? Si No N/P

20. Il bambino respira rumorosamente con il naso e/o respira a fatica durante il sonno

Di solito (5-7)☐ Qualche volta (2-4)☐ Raramente (0-1) ☐ Problema? Si No N/P

21. Il bambino ha problemi a dormire fuori casa (a casa di parenti, in vacanza)

Di solito (5-7)☐ Qualche volta (2-4)☐ Raramente (0-1) ☐ Problema? Si No N/P

22. Il bambino si sveglia durante la notte urlando, sudato e inconsolabile

Di solito (5-7)☐ Qualche volta (2-4)☐ Raramente (0-1) ☐ Problema? Si No N/P

23. Il bambino si sveglia agitato da un sogno spaventoso

Di solito (5-7)☐ Qualche volta (2-4)☐ Raramente (0-1) ☐ Problema? Si No N/P

Risvegli durante la notte

24. Il bambino si sveglia una volta sola durante la notte

Di solito (5-7)☐ Qualche volta (2-4)☐ Raramente (0-1) ☐ Problema? Si No N/P

25. Il bambino si sveglia più di una volta durante la notte

Di solito (5-7)☐ Qualche volta (2-4)☐ Raramente (0-1) ☐ Problema? Si No N/P

Riportare quanto dura di solito il risveglio notturno, in minuti______

Risveglio al mattino/Sonnolenza durante il giorno

Riporta a che ora di solito il bambino si sveglia al mattino______

26. Il bambino si sveglia da solo

Di solito (5-7)☐ Qualche volta (2-4)☐ Raramente (0-1) ☐ Problema? Si No N/P

27. Il bambino si sveglia di cattivo umore

Di solito (5-7)☐ Qualche volta (2-4)☐ Raramente (0-1) ☐ Problema? Si No N/P

28. Adulti o fratelli svegliano il bambino

Di solito (5-7)☐ Qualche volta (2-4)☐ Raramente (0-1) ☐ Problema? Si No N/P

29. Il bambino ha difficoltà ad alzarsi dal letto al mattino

Di solito (5-7)☐ Qualche volta (2-4)☐ Raramente (0-1) ☐ Problema? Si No N/P

30. Il bambino impiega molto tempo ad essere completamente sveglio al mattino

Di solito (5-7)☐ Qualche volta (2-4)☐ Raramente (0-1) ☐ Problema? Si No N/P

31. Il bambino sembra stanco

Di solito (5-7)☐ Qualche volta (2-4)☐ Raramente (0-1) ☐ Problema? Si No N/P

Il bambino appare molto assonnato o si addormenta nelle seguenti condizioni (segni tutte quelle pertinenti)

32. Guardando la TV

Non sonnolento☐ Molto sonnolento☐ Si addormenta☐

33. Durante un tragitto in auto

Non sonnolento☐ Molto sonnolento☐ Si addormenta☐
