# Supplementary material for: Obstructive Sleep Apnoea in Children with Down Syndrome: A Multidisciplinary Approach
Source: J Pers Med. 2022 Dec 28;13(1):71. doi: 10.3390/jpm13010071 (PMC9862921; doi:10.3390/jpm13010071)
Supplement: Supplementary file 1 [file jpm-13-00071-s001.zip › Table S1.pdf]

Table S1. Comparison of total and subscales scores of CSHQ-IT administered to mothers of healthy children and Down syndrome.

	Healthy children (n=48)	Down syndrome children (n=48)	p
**Total score**	45.8 ± 6.9	63 ± 5.1	0.001
**Bedtime resistance** (items 1-3-4-5-6-8)	8.8 ± 2.6	11.3 ± 3.3	0.001
**Sleep onset delay** (item 2)	1.4 ± 0.7	1.4 ± 0.7	0.93
**Sleep anxiety** (5-7-8-21)	6.2 ± 2.2	7.3 ± 2.2	0.017
**Sleep duration** (items 9-10-11)	3.6 ± 1.2	4.6 ± 1.6	0.007
**Night wakings** (items 16-24-25)	4 ± 1.1	4.8 ± 1.6	0.008
**Parasomnias** (items 12-13-14-15-17-22-23)	8.8 ± 1.9	9.8 ± 1.8	0.013
**Sleep-disordered breathing** (items 18-19-20)	3.4 ± 0.7	4.4 ± 1.7	0.002
**Day time sleepiness** (items 26-27-28-29-30-31-32-33)	12.9 ± 2.8	14.5 ± 3.5	0.025

Data are expressed as mean ± standard deviation. The Student t- test was performed.

*$p<0.05$.

Abbreviations: CSHQ-IT, Children's Sleep Habits Questionnaire adapted to Italian language [12, 13].
