# Supplementary material for: Obstructive Sleep Apnoea in Children with Down Syndrome: A Multidisciplinary Approach
Source: J Pers Med. 2022 Dec 28;13(1):71. doi: 10.3390/jpm13010071 (PMC9862921; doi:10.3390/jpm13010071)
Supplement: Supplementary file 1 [file jpm-13-00071-s001.zip › Table S2.pdf]

Table S2. CSHQ-IT total and subscale scores in children with Down syndrome with mild or moderate/severe OSA or without OSA.

	OSA			OSA severity		
	**Yes (n=32)**	**No (n=16)**	**p**	**Mild (n=20)**	**Moderate/severe (n=12)**	**p**
**Total score**	57.3±9	53.6±8.8	0.172	56.4±9.5	55.1±7.6	0.625
**Bedtime resistance** (items 1-3-4-5-6-8)	12.2±2.9	11.6±3.1	0.483	12.2±3	11.2±2.9	0.315
**Sleep onset delay** (item 2)	1.4±0.8	1.5±0.7	0.785	1.4±0.7	1.6±0.8	0.529
**Sleep anxiety** (items 5-7-8-21)	7.9±2.2	7.7±2	0.734	7.9±2.2	7.6±2.1	0.643
**Sleep duration** (items 9-10-11)	4.5±1.9	4.2±1.6	0.56	4.4±-2	4.3±1.3	0.868
**Night wakings** (items 16-24-25)	5.2±1.7	4.7±1.4	0.305	5.2±1.6	4.4±1.6	0.152
**Parasomnias** (items 12-13-14-15-17-22-23)	10.3±1.6	10.2±1.6	0.85	10.4±1.7	9.8±1.1	0.206
**Sleep-disordered breathing** (items 18-19-20)	5.6±2	4.8±1.7	0.184	5.2±1.8	5.8±2.1	0.409
**Day time sleepiness** (items 26-27-28-29-30-31-32-33)	14.9±4.1	13.4±3.6	0.223	14.3±4	14.8±4.1	0.731

Data are expressed as mean ± standard deviation. The Student t- test was performed.

*p<0.05.

Abbreviations: CSHQ-IT, Children's Sleep Habits Questionnaire in Italian; OSA, Obstructive Sleep Apnea, AHI>1; No OSA, AHI ≤1; Mild OSA, AHI >1 and ≤5; Moderate/severe OSA, AHI > 5.
