# Supplementary material for: Obstructive Sleep Apnoea in Children with Down Syndrome: A Multidisciplinary Approach
Source: J Pers Med. 2022 Dec 28;13(1):71. doi: 10.3390/jpm13010071 (PMC9862921; doi:10.3390/jpm13010071)
Supplement: Supplementary file 1 [file jpm-13-00071-s001.zip › Table S3.pdf]

Table S3. Ear Nose Throat evaluation in children with Down syndrome with mild or moderate/severe OSA or without OSA.

		OSA			OSAS severity		
		**Yes (n=21)**	**No (n=13)**	**p**	**Mild (n=11)**	**Moderate/severe (n=10)**	**p**
**Palatine tonsils hypertrophy size**	Size 1-2	7	6	0.5	3	4	0.6
	Size 3-4	14	7		8	6	
**Adenoid tonsils hypertrophy size**	I-II grade	10	9	0.3	7	3	0.2
	III grade	11	4		4	7	
**Chronic rhinosinusitis score**	Yes (>2)	18	11	1	10	8	0.6
	No (≤2)	3	2		1	2	
**Nasal turbinate hypertrophy**	Present	14	9	1	7	7	1
	Absent	7	4		4	3	
**Nasal septum deviation**	Present	11	6	1	5	6	0.7
	Absent	10	7		6	4	

The Fisher exact test was performed.

*$p<0.05$.

Abbreviations: OSA, Obstructive Sleep Apnea, AHI>1; No OSA, AHI ≤1; Mild OSA, AHI >1 and ≤5; Moderate/severe OSA, AHI > 5.
