# Supplementary material for: Obstructive Sleep Apnoea in Children with Down Syndrome: A Multidisciplinary Approach
Source: J Pers Med. 2022 Dec 28;13(1):71. doi: 10.3390/jpm13010071 (PMC9862921; doi:10.3390/jpm13010071)
Supplement: Supplementary file 1 [file jpm-13-00071-s001.zip › Table S4.pdf]

Table S4. Orthodontic evaluation in children with Down syndrome with mild or moderate/severe OSA or without OSA.

		OSA			OSA severity		
		**Yes (n= 31)**	**No (n= 16)**	**p**	**Mild (n=19)**	**Moderate/severe (n=12)**	**p**
**Facial profile**	Orthognathic	21	11	0.304	14	7	0.180
	Concave	8	2		3	5	
	Convex	2	3		2	0	
**Molar class relationship**	I	18	6	0.058	13	5	0.074
	II	3	3		1	2	
	III	7	1		5	2	
	NA	3	6		0	3	
**Overbite**	Normal	27	11	0.194	17	10	0.630
	Open bite	4	4		2	2	
	Deep bite	0	1		0	0	
**Cross-bite**	Present	15	8	1	8	7	0.473
	Absent	16	8		11	5	
**Bruxism**	Present	14	8	0.768	8	6	0.724
	Absent	17	8		11	6	
**Oral parafunctional behaviours**	Present	7	7	0.182	3	4	0.384
	Absent	24	9		16	8	

The X^2-test was performed.

*$p<0.05$.

Abbreviations: NA, not available as first molars were not present; OSA, Obstructive Sleep Apnea, AHI>1; No OSA, AHI ≤1; Mild OSA, AHI >1 and ≤5; Moderate/severe OSA, AHI > 5.
